# Supplementary material for: Synergistic Antifungal Effect and In Vivo Toxicity of a Monoterpene Isoespintanol Obtained from Oxandra xylopioides Diels
Source: Molecules. 2024 Sep 17;29(18):4417. doi: 10.3390/molecules29184417 (PMC11433975; doi:10.3390/molecules29184417)
Supplement: Supplementary file 1 [file molecules-29-04417-s001.zip › molecules-3187893-supplementary.pdf]

Article

Synergistic Antifungal Effect and In Vivo Toxicity of a Monoterpene Isoespintanol Obtained from *Oxandra xylopioides* Diels

Orfa Inés Contreras-Martínez [1], Alberto Angulo-Ortíz [2,*], Gilmar Santafé Patiño [2], Jesus Sierra Martinez [3], Ricardo Berrio Soto [1], Joice Margareth de Almeida Rodolpho [4], Krissia Franco de Godoy [4], Fernanda de Freitas Aníbal [4] and Bruna Dias de Lima Fragelli [5]

[1] Biology Department, Faculty of Basic Sciences, University of Córdoba, Montería 230002, Colombia; oicontreras@correo.unicordoba.edu.co (O.I.C.-M.); rberriosoto54@correo.unicordoba.edu.co (R.B.S.)
[2] Chemistry Department, Faculty of Basic Sciences, University of Córdoba, Montería 230002, Colombia; gsantafe@correo.unicordoba.edu.co
[3] Department of Genetics and Evolution, Federal University of São Carlos, São Carlos 13565-905, SP, Brazil; jesussierra@estudante.ufscar.br
[4] Laboratory of Inflammation and Infectious Diseases, Department of Morphology and Pathology, Federal University of São Carlos, São Carlos 13565-905, SP, Brazil; j_jrodolpho@hotmail.com (J.M.d.A.R.); krissia.fgodoy@gmail.com (K.F.d.G.); ffanibal@ufscar.br (F.d.F.A.)
[5] Functional Materials Development Center, Interdisciplinary Laboratory of Electrochemistry and Ceramics, Department of Chemistry, Federal University of São Carlos, São Carlos 13565-905, SP, Brazil; brufragelli@gmail.com
* Correspondence: aaangulo@correo.unicordoba.edu.co

Supplementary Materials

Table S1: Histopathological report of the acute phase. After exposure to the compound Isoespintanol, the liver, lungs and kidneys of all groups were analyzed: G1: CTRL + water; G2: CTRL + oil; G3 - G6: 25, 50, 100 and 200 mg/ml of Isoespintanol after 14 days.

GROUP	ORGAN	MICROSCOPIC FINDINGS
Water Control (G1)	Liver (A)	Moderately swollen hepatocytes, diffuse to a moderate degree; presence of binucleated hepatocytes (sign of regeneration); there was a discreet perivascular lymphoplasmocytic inflammatory infiltrate (around the triad).
	Lungs (B)	Presence of slight hemorrhage in the lung parenchyma.
	Kidneys (C)	No microscopic changes of note.
Oil control (G2)	Liver (A)	Moderately swollen hepatocytes, diffuse to a moderate degree; presence of binucleated hepatocytes (sign of regeneration).
	Lungs (B)	Presence of discrete hemorrhagic points in the pulmonary interstitium (multifocal).
	Kidneys (C)	No microscopic changes of note.
25mg (G3)	Liver (A)	Moderately swollen hepatocytes, diffuse to a moderate degree; presence of binucleated hepatocytes (sign of regeneration); there was a slight lymphoplasmacytic inflammatory infiltrate in small clusters in the parenchyma.

	Lungs (B)	Presence of small and multiple foci of hemorrhage in the interstitium.
	Kidneys (C)	Presence of discrete foci of lymphoplasmocytic infiltrate in the cortical zone, close to the glomeruli.
50 mg (G4)	Liver (A)	Moderately swollen hepatocytes, diffuse to a moderate degree; presence of binucleated hepatocytes (sign of regeneration), as well as discrete foci of necrosis. Presence of discrete foci of peripheral perivascular lymphoplasmocytic infiltrate and in the hepatic interstitium.
	Lungs (B)	Presence of focally extensive areas of hemorrhage in the pulmonary interstitium.
	Kidneys (C)	Presence of discrete foci of lymphoplasmocytic infiltrate in the cortical and medullary zones.
100 mg (G5)	Liver (A)	Moderately swollen hepatocytes, diffuse to a moderate degree; presence of binucleated hepatocytes (sign of regeneration), as well as discrete foci of necrosis. Presence of discrete foci of lymphoplasmocytic lymphoplasmocytic infiltrate in the hepatic interstitium. Hepatocyte nuclei markedly evident.
	Lungs (B)	Presence of moderate thickening of the alveolar septa due to a multifocal and moderate multifocal and moderate lymphoplasmocytic infiltrate around the bronchi, bronchioles and in the alveoli
	Kidneys (C)	Presence of discrete foci of lymphoplasmocytic infiltrate in the cortical zone, close to the glomeruli.
200 mg (G6)	Liver (A)	Moderately swollen hepatocytes, diffuse to a moderate degree; presence of binucleated hepatocytes (sign of regeneration), as well as discrete foci of necrosis.
	Lungs (B)	There was a thickening of the alveolar septa (diffuse and to a moderate degree) due to a moderate and diffuse inflammatory infiltrate.
	Kidneys (C)	No microscopic changes of note.

Table S2: Histopathological report of the chronic phase. After exposure to the compound Isoespintanol, the liver, lungs and kidneys of all groups were analyzed: G1: CTRL + water; G2: CTRL + oil; G3 - G6: 25, 50, 100 and 200 mg/ml of Isoespintanol after 98 days.

GROUP	ORGAN	MICROSCOPIC FINDINGS
Water Control (G1)	Liver (A)	Moderately swollen hepatocytes, diffuse to a moderate degree; presence of binucleated hepatocytes (sign of regeneration), as well as discrete foci of necrosis.
	Lungs (B)	There was a thickening of the alveolar septa (diffuse and to a moderate degree) because of an infiltrated moderate and diffuse lymphoplasmocytic inflammatory infiltrate
	Kidneys (C)	Presence of extensive foci of lymphoplasmocytic infiltrate in the cortical and medullary zones
Oil control (G2)	Liver (A)	Presence of multiple foci of mixed inflammatory infiltrate (lymphocytes, plasma cells and neutrophils), in moderate degree moderate degree and in the middle of the hepatocytes. There were areas of granuloma (with the presence of giant perivascular and in the parenchyma, not encapsulated, but with a rounded conformation.
	Lungs (B)	Presence of focally extensive areas of hemorrhage in the pulmonary interstitium, slight degree.
	Kidneys (C)	No microscopic changes of note.
25 mg (G3)	Liver (A)	Presence of small foci of perivascular and free lymphoplasmocytic infiltrate in the liver parenchyma

	Lungs (B)	Presence of small hemorrhagic foci in the interstitium, associated with a slight thickening of the alveolar septa alveolar septa by a discreet diffuse lymphoplasmocytic infiltrate.
	Kidneys (C)	Presence of multiple foci of moderate lymphoplasmocytic infiltrate in the cortical zone.
50 mg (G4)	Liver (A)	Presence of small foci of lymphoplasmocytic inflammatory infiltrate in the parenchyma and perivascular area.
	Lungs (B)	Marked evidence of peri-bronchial B.A.L.T; multifocal areas of thickening of the alveolar septa alveolar septa by lymphoplasmocytic inflammatory infiltrate.
	Kidneys (C)	Presence of multiple foci of moderate lymphoplasmocytic infiltrate in the cortical zone.
100 mg (G5)	Liver (A)	Hepatocytes diffusely swollen; presence of binucleated hepatocytes (sign of regeneration).
	Lungs (B)	Moderately thickened alveolar septa due to moderate and diffuse lymphoplasmocytic infiltrate.
	Kidneys (C)	Presence of multiple foci of mild lymphoplasmocytic infiltrate in the cortical zone.
200 mg (G6)	Liver (A)	Presence of multiple foci of mixed inflammatory infiltrate (lymphocytes, plasma cells and neutrophils), in moderate degree moderate degree and in the middle of the hepatocytes. There were areas of granuloma (with the presence of giant perivascular and in the parenchyma, not encapsulated, but with a rounded conformation.
	Lungs (B)	Presence of focally extensive areas of hemorrhage in the pulmonary interstitium, slight degree.
	Kidneys (C)	Presence of multiple foci of lymphoplasmocytic infiltrate, to a slight degree, in the cortical zone.
